# A nomogram model for early recurrence of HBV-related hepatocellular carcinomas after radical hepatectomy

**DOI:** 10.3389/fendo.2024.1374245

**Published:** 2024-09-02

**Authors:** Yu Zhu, Ai-Dong Wang, Ling-Ling Gu, Qi-Qiang Dai, Guo-Qun Zheng, Ting Chen, Chun-Long Wu, Wei-Dong Jia, Fa-Biao Zhang

**Affiliations:** ^1^ Department of Hepatopancreatobiliary Surgery, Taizhou Hospital of Zhejiang Province Affiliated to Wenzhou Medical University, Taizhou, China; ^2^ Division of Liver Surgery, The First Affiliated Hospital of University of Science and Technology of China (USTC), Division of Life Sciences and Medicine, University of Science and Technology of China, Hefei, China; ^3^ Department of Hepatopancreatobiliary Surgery, Enze Hospital of Taizhou, Taizhou, China

**Keywords:** hepatocellular carcinoma, risk factors, radical hepatectomy, early recurrence, nomogram

## Abstract

**Background:**

To identify the risk factors and construct a predictive model for early recurrence of hepatitis B virus(HBV-)- related hepatocellular carcinomas(HCCs) after radical resection.

**Data and methods:**

A total of 465 HBV-related HCC patients underwent radical resections between January 1, 2012 and August 31, 2018.Their data were collected through the inpatient information management system of the First Affiliated Hospital of University of Science and Technology of China. Survival and subgroup analyses of early recurrence among male and female patients were performed using Kaplan-Meier curves. The independent risk factors associated with early postoperative tumor recurrence were analyzed using multivariate Cox proportional hazards regression model. Based on these independent risk factors, a risk function model for early recurrence was fitted, and a column chart for the prediction model was drawn for internal and external validation.

**Results:**

A total of 181 patients developed early recurrences, including 156 males and 25 females. There was no difference in the early recurrence rates between males and females. Tumor diameters>5cm, microvascular invasion and albumin level<35 g/L were independent risk factors for early recurrence. A nomogram for the early recurrence prediction model was drawn; the areas under the curve for the model and for external verification were 0.638 and 0.655, respectively.

**Conclusion:**

Tumor diameter>5 cm, microvascular invasion, and albumin level<35 g/L were independent risk factors for early recurrence. The prediction model based on three clinical indicators could predict early recurrence, with good discrimination, calibration, and extrapolation.

## Introduction

Cancer is the second most common cause of death, in humans, while liver cancer is the fourth most common cause of cancer-related deaths (1). Approximately 85,000 new liver cancer cases occur globally every year (2).In China, the incidence and mortality rates for liver cancer are 28.12 and 24.33 per 100,000,respectively (3). Hepatocellular carcinoma (HCC) is the most common pathological type of liver cancer (4),accounting for 75-85% of all liver cancers (1). HCCs have the fourth highest incidence rate and second highest mortality rate among all cancer types in China (5). The overall survival rate(OS) after hepatectomy remains unsatisfactory. The 5-year survival rate for HCC in the United States is only 21% (6), while the 5-year recurrence rate after liver resection for HCCs is as high as 50-70% (4, 7). Early postoperative tumor recurrence can lead to poor prognosis; therefore, taking measures to reduce the recurrence rate and improve overall survival is crucial in these patients (8, 9). The present study identified the important risk factors for early recurrence after curative resection of hepatitis B virus(HBV-)-related HCCs, and built an early recurrence prediction model, thus, we will facilitate the development of effective intervention measures to prevent or delay HCC early recurrence and improving the long team survival of HCC patients.

## Materials and methods

### Study participants

**Figure 1:**
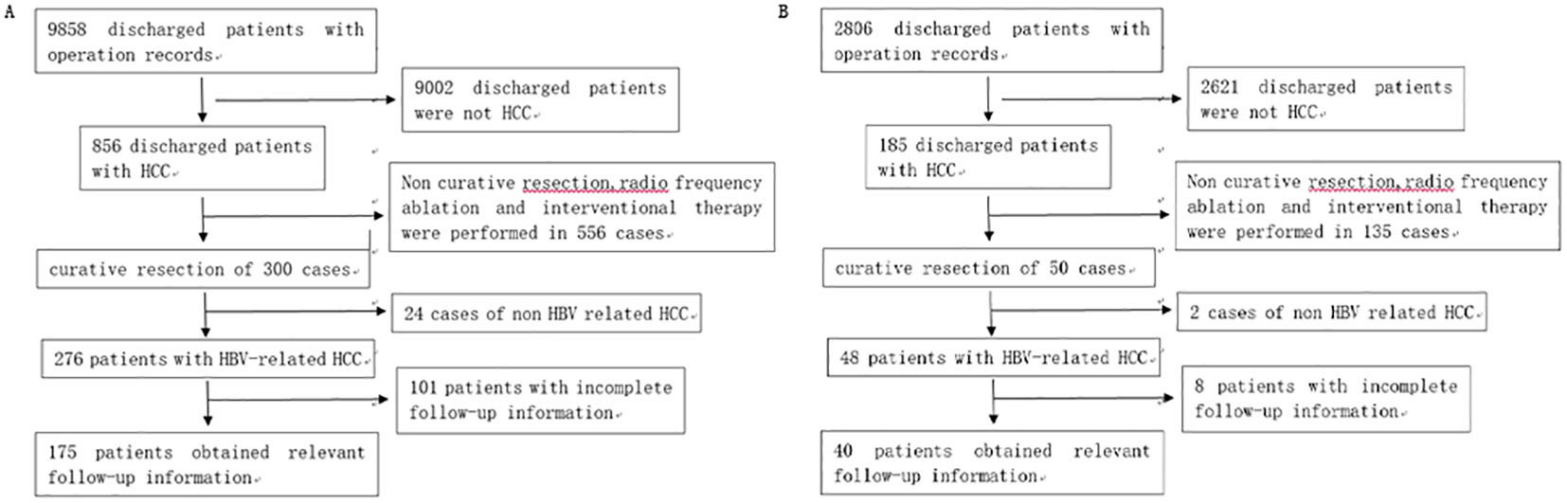
**(A)** Patients’ selection process at Taizhou Hospital affiliated with Wenzhou Medical University. **(B)** Patients’ selection process at Enze Hospital of Taizhou.

We collected the data for 3,958 patients, treated for HCC at the First Affiliated Hospital of University of Science and Technology of China between January 1, 2012 and August 31, 2018, from the inpatient information management system. The inclusion/and exclusion criteria and the screening process are detailed in our previous study (10). Based on the inclusion/exclusion criteria, a total of 465 patients were included in the training set. We also collected the data for 175 patients from Taizhou Hospital affiliated with Wenzhou Medical University between January 1, 2012 and August 31, 2018, and 40 patients from Enze Hospital of Taizhou between January 1, 2015 and August 31, 2018 as the external validation set. The screening processes are shown in **Figure 1**.

### Data collection

Data were collected for the following factors that could potentially influence early recurrence.

(1) Basic factors: age, sex, drinking and smoking history, Child-Pugh classification, cirrhosis, and ascites.
(2) Preoperative serum factors: alanine aminotransferase, total bilirubin, albumin, hepatitis B core antibody, hepatitis B surface antigen, HBV-DNA, alpha fetoprotein, prothrombin time, platelets, neutrophil-to-lymphocyte ratio, serum albumin-to-globulin ratio, and platelet-to-lymphocyte ratio.
(3) Intraoperative factors: intraoperative bleeding, surgical approaches, perioperative blood transfusion, hepatectomy type, and surgical duration.
(4) Tumor-related factors: tumor size and number, capsule integrity, microvascular invasion (MVI), Edmondson grade, Chinese Liver Cancer stage (2019), and Barcelona Clinic Liver Cancer stage (2018).

We collected information about tumor recurrence and recurrence time through the telephone and electronic information system; the recurrence time was accurate to month. The starting point for the follow-up was date of surgery, while the endpoint was patient’s death or December 31, 2019. All patients were given anti hepatitis B virus treatment after surgery, antiviral drugs include lamivudine, entecavir, and adefovir.

### Statistical analysis

Statistical analyses were performed using R 4.1.0 and SAS 9.4. Measurement data with normal or approximately normal distributions were analyzed as x ± s, and the groups were compared using a *t*-test; M (P25 ∼ P75) was used to describe non-normally distributed measurement data; the non-parametric rank sum test was used for inter-group comparisons. Categorical variables were presented as frequencies or numbers and percentages. The groups were compared using χ 2 test; Early recurrence rates were calculated using the Kaplan-Meier method, and the recurrence-free survival curve was plotted. The independent risk factors for early recurrence were analyzed using the multivariate Cox proportional risk regression model.

The recurrence prediction model was established using the independent risk factors identified in the multifactor Cox proportional risk regression model, Impact intensities for the related factors, along with the hazard ratios and 95% confidence intercals (CI), were estimated. A receiver operating characteristics(ROC) curve was drawn to evaluate the prediction effects of the model. A nomogram was used to display the prediction model, with a threshold α level of 0.05. The Bootstrap resampling method was used for internal validation, with 1000 repeated samples. The external validation data were used to verify the extrapolation of the prediction model, evaluate its prediction accuracy (area under the ROC curve), and calibrate it using the calibration curve.

## Results

**Figure 2:**
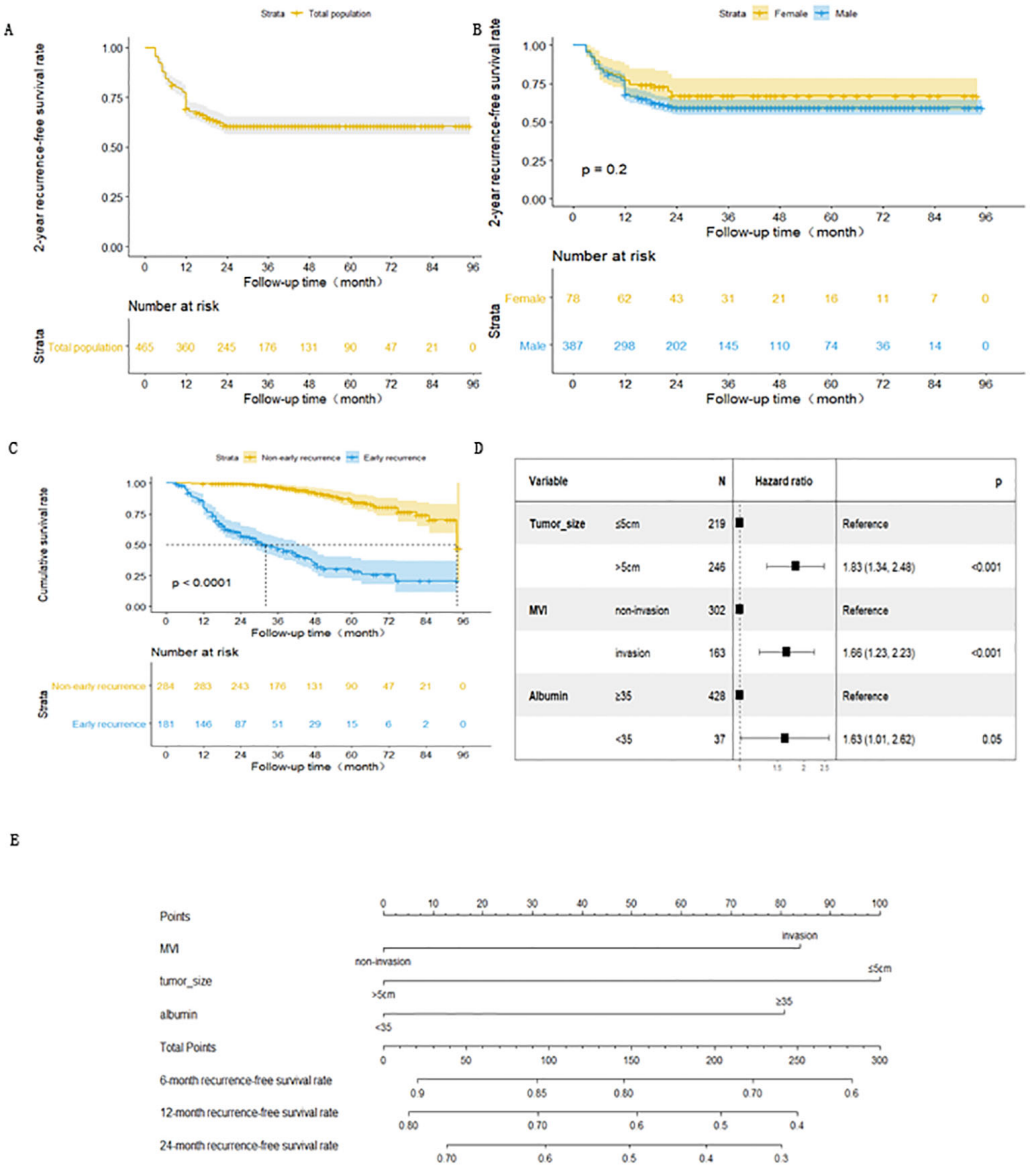
**(A)** Kaplan-Meiers curve for early recurrence in HCC patients after radical resection. **(B)** Kaplan-Meiers curve for early recurrence of HCC in male and female patients after radical resection. **(C)** Survival curve of patients with HBV-related HCC non-early recurrence group and early recurrence group after radical resection. **(D)** Forest map of risk factors for early recurrence of HBV- related HCC patients after radical resection. **(E)** Nomogram of early recurrence prediction model for HBV-related HCC patients after radical resection.

The study included 465 patients with HBV-related HCCs who underwent radical resections. There were no perioperative deaths. **Table 1** shows the basic characteristics of the study participants with and without early postoperative recurrence. The patients were followed up for a median of 26(3-95) months. A total of 181 HCC patients developed early tumor recurrences after radical resection. The 1- and 2-year recurrence- free survival rates were 69.4% and 60.6%, respectively. **Figure 2A** shows the Kaplan Meier survival curve for early recurrence of HBV-related HCCs after curative resection.

**Table 1:** Basic characteristics of early recurrence and non early recurrence in patients with hepatitis B-related HCC (n = 465).

basic characteristics	non early recurrence	early recurrence	*P*
Age(years)	56.89 ± 11.56	55.67 ± 11.66	0.271
Sex[number of cases (%)]			0.172
female/male	53(18.7)/231(81.3)	25(13.8)/156(86.2)	
Drinking [number of cases (%)]			0.211
none/yes	227(79.9)/57(20.1)	153(84.5)/28(15.5)	
Smoking[number of cases (%)]			0.746
none/yes	200(70.4)/84(29.6)	130(71.8)/51(28.2)	
NLR	1.89(1.49-2.62)	2.32(1.69-3.25)	0.025
PLR	92.7(68.8-126.6)	96.4(74.5-136.7)	0.086
AGR	1.43 ± 0.27	1.38 ± 0.26	0.042
Intraoperative bleeding(ml)	200(100-300)	200(100-500)	0.675
Tumor size [number of cases (%)]			<.000
≤5/>5cm	155(54.6)/129(45.4)	64(35.4)/117(64.6)	
Cirrhosis [number of cases (%)]			0.883
none/yes	66(23.2)/218(76.8)	41(22.7)/140(77.3)	
MVI[number of cases (%)]			<.000
none/yes	203(71.5)/81(28.5)	99(54.7)/82(45.3)	
Tumor number [number of cases (%)]			0.118
1/≥2	254(89.4)/30(10.6)	153(84.5)/28(15.5)	
Intact capsule [number of cases (%)]			0.033
none/yes	49(17.2)/235(82.8)	46(25.4)/135(74.6)	
Anatomical resection [number of cases (%)]			0.043
none/yes	220(77.5)/64(22.5)	125(69.1)/56(30.9)	
AFP[number of cases (%)]			0.621
<400/≥400ng/ml	193(68.0)/91(32.0)	119(65.8)/62(34.2)	
Edmondson[number of cases (%)]			0.034
I and II/III and IV	214(75.4)/70(24.6)	120(66.3)/61(33.7)	
Anti-HBc [number of cases (%)]			0.126
positive/negative	272(95.8)/12(4.2)	178(98.3)/3(1.7)	
HBsAg [number of cases (%)]			0.787
positive/negative	239(84.2)/45(15.8)	154(85.1)/27(14.9)	
HBV-DNA[number of cases (%)]			0.039
<1000/≥1000copies/ml	118(41.6)/166(58.4)	58(32.0)/123(68.0)	
ALT[number of cases (%)]			0.157
≤50/>50 U/L	219(77.1)/65(22.9)	129(71.3)/52(28.7)	
ALB[number of cases (%)]			0.006
≥35/<35 g/L	266(93.7)/18(6.3)	162(89.5)/19(10.5)	
TB[number of cases (%)]			0.662
≤20.5/>20.5μmol/L	223(78.5)/61(21.5)	139(76.8)/42(23.2)	
PT[number of cases (%)]			0.558
≤14/>14 s	261(91.9)/23(8.1)	169(93.4)/12(6.6)	
Ascites [number of cases (%)]			0.740
none/yes	240(84.5)/44(15.5)	155(85.6)/26(14.4)	
Perioperative blood transfusion [number of cases (%)]			0.013
none/yes	241(84.9)/43(15.1)	137(75.7)/44(24.3)	
hepatectomy type [number of cases (%)]			0.653
Laparoscopy/Laparotomy	32(11.3)/252(88.7)	18(9.9)/163(90.1)	
Operation duration [number of cases (%)]			0.208
≥120/<120 min	151(53.2)/133(46.8)	107(59.1)/74(40.9)	
Child-Pugh[number of cases (%)]			0.748
A/B	283(99.7)/1(0.3)	180(99.5)/1(0.5)	
CNLC[number of cases (%)]			0.483
Ia/Ib/IIa/IIb	173(60.9)/84(29.6)/23(8.1)/4(1.4)	67(37.0)/88(48.6)/22(12.2)/4(2.2)	
BCLC[number of cases (%)]			0.059
0/A/B	22(7.8)/240(84.5)/22(7.7)	8(4.4)/148(81.8)/25(13.8)	

NLR, neutrophil-to-lymphocyte ratio; PLR, platelet-to-lymphocyte ratio; AGR, serum albumin-to-globulin ratio; MVI, microvascular invasion; ALT, alanine aminotransferase; ALB, albumin; HBsAg, hepatitis B surface antigen; Anti-HBc, hepatitis B core antibody; AFP, alpha fetoprotein; TB, total bilirubin; PT, prothrombin time; CNLC, Chinese Liver Cancer stage; BCLC, Barcelona Clinic Liver Cancer stage.

There were 156 and 25 cases of early recurrences among males and females, respectively. The 1-year recurrence-free survival rates for male and females were 67.9% and 76.9%, respectively. There was no statistically significant difference in the recurrence-free survival curve between males and females (**Figure 2B**).

A total of 31 non-early recurrence group patients died, with a median survival time of 94 months. Meanwhile, 101 early recurrence group patients died, with a median survival time of 31 months. The cumulative survival rates at 1, 3, and 5 years after surgery were 99.6%, 96.5% and 85.3% for the non-early recurrence group and 79.6%, 46.7%, and 25.4% for the early recurrence group, respectively. There was a statistically significant difference in survival curves between the groups(**Figure 2C**).

The survival outcome (early recurrence group) and relapse-free survival time were taken as dependent variables, and each related factor was taken as an independent variable to conduct multivariate Cox proportional hazard regression model analysis (variable screening method STEPWISE, variable inclusion criteria α=0.05, and a removal standard of 0.10). The independent risk factors for early recurrence of HBV-related HCC patients were tumor diameter >5 cm, albumin level <35 g/L, and MVI (**Table 2** and **Figure 2D**).

**Table 2:** Multivariate COX hazard model for early recurrence of HBV related HCC patients undergoing curative resection HR (95%CI).

Risk factors	n	HR(95%CI)	c2	*P*
Tumor size				
≤5 cm	219	1	13.89	0.0002
>5 cm	246	1.83(1.34-2.48)		
MVI				
None	302	1	10.71	0.0011
Yes	163	1.66(1.23-2.23)		
ALB				
≥35 g/L	428	1		
<35 g/L	37	1.63(1.01-2.62)	10.71	0.0011

Based on these results, the expression of the early recurrence risk function model for HBV-related HCCs after curative resection was fitted as follows: h(t) = h0exp(0.60X1 + 0.50X2 + 0.48X3). X1, X2, and X3 are taken as 1 if tumor diameter is ≤ 5 cm, MVI is present, and albumin index is <35 g/L, respectively. Otherwise, a value of 0 is assigned to them. The likelihood ratio test was conducted on the prediction model, and the difference was statistically significant (χ 2 = 32.23, *p* < 0.001). To allow a more intuitive presentation and easier clinical application of the prediction model, the model predictions were presented in the form of a nomogram (**Figure 2E**).

**Figure 3:**
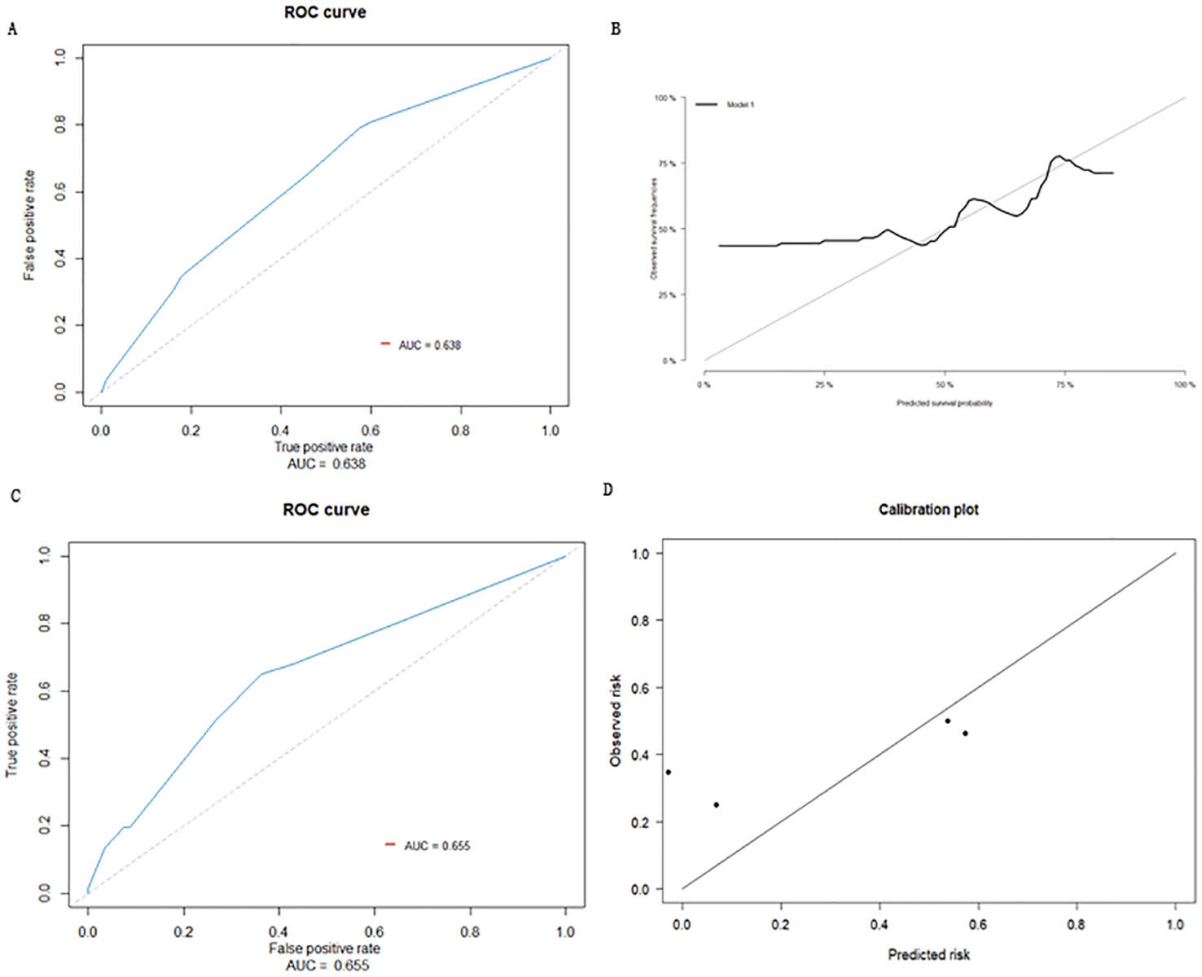
**(A)** ROC curve of prediction model. **(B)** Calibration curve of internal validation population of early recurrence prediction model. **(C)** External inspection ROC curve of early recurrence prediction model. **(D)** Calibration curve of external validation population of early recurrence prediction model.

The prediction effect was evaluated using the area under the curve (AUC) for the ROC curve. The prediction effect of the prediction model ROC is shown in **Figure 3A**, with AUC = 0.638 (95% CI: 0.588-0.687). Internal validation was conducted by using the Bootstrap method, and cross validation was performed using the Bootstrap resampling method. The model development queue was used as the validation set to evaluate the model performance. This process was repeated 1000 times; the final calibration curve obtained is shown in **Figure 3B**.

Based on the data for 215 cases, collected from Taizhou Hospital affiliated with Wenzhou Medical University and Taizhou Enze Hospital as external datasets, 66 HCC patients who underwent radical resections developed early recurrences, while 149 did not. Three sets of data for MVI, tumor diameter, and serum albumin level were obtained from these cases for external validation of the model. The prediction accuracy of the model was evaluated as the AUC for the ROC curve; the AUC for the external validation population was predicted to be 0.655 (95% CI: 0.579-0.732) (**Figure 3C**). The prediction model was distinguishable, indicating that the model can be extrapolated to a certain degree. The calibration curve showed that the prediction probability of the model was close to the actual observed probability, with a certain degree of consistency (**Figure 3D**).

## Discussion

Despite several recent improvements in HCC treatment, including the advancements in the surgical technology and development of systemic therapeutic drugs, the overall survival duration remains unsatisfactory. The patients are at risk of a recurrence even after curative resection (11). The pathophysiology and mechanism of recurrence and metastasis in HCCs after radical resection have not been fully elucidated. The major oncogenic molecular signaling pathways in HCC, including receptor tyrosine kinases (RTKs), RAS/RAF/MEK/extracellular‐signal‐regulated kinase(ERK), phosphoinositide 3‐kinase (PI3K)/AKT/mammalian target of rapamycin (mTOR), Wnt/β‐catenin, janus‐kinase (JAK)/signal transducer activator of transcription (STAT), Hedgehog (Hh), Hippo pathway (12). In recent years, targeted and immunotherapy have made rapid progress, and molecules that affect HCC recurrence and metastasis have been continuously discovered, such as, Trigger receptor expressed on myeloid cells-2(TREM2) deficiency increased the therapeutic effect of anti-programmed death ligand-1(PD-L1) blockade by enhancing antitumor activity of CD8+ T cells (13), mitochondrial transcription factor A(TFAM) may serve as an effective target to block HCC metastasis. These findings provide new target for HCC immunotherapy (14). However, these are complex processes involving several inter-related factors. Effective reduction of HCC recurrence after surgery and increasing the survival duration are constantly pursued goals. Therefore, there is an urgent need to develop newer predictive methods to evaluate HCC recurrence rates after surgery.

Currently, there is no standard definition for early HCC recurrence after surgery. The time for early recurrence, defined in various studies, ranges from 6 months to 2 years (15–17). It is believed that there are two sources of recurrent cancer cells, tumor cells derived from the residual tumor after hepatectomy (single center origin) and those emerging in the liver after hepatectomy (multi center origin). It is generally believed that HCC recurrence within 2 years after the surgery are mainly caused by residual tumor cells, which are mostly related to tumor characteristics, including diameter, invasiveness, and serum alpha fetoprotein levels. This mainly manifests as local infiltration of the primary focus and subclinical dissemination in the liver. Meanwhile, recurrences that occur beyond 2 years after the surgery are mostly caused by new tumor cells in the liver after hepatectomy, which may be related to chronic liver inflammation and cirrhosis (18, 19). These sources of tumor cells are not addressed by hepatectomy alone, and the tumor may recur in the presence of inflammation or cirrhosis. It can be seen that the mechanisms of recurrence within and after 2 years are different. Early recurrence is a crucial factor affecting the survival rate of HCC patients after surgery, and it can lead to poor prognosis after radical hepatectomy (15), Therefore, it is important to analyze the risk factors for early recurrence after surgery. The present study defined HCC recurrence within 2 years after surgery as early recurrence, including intrahepatic recurrences and extrahepatic metastases.

Early postoperative recurrence of HCC is a complex process, which occurs over multiple steps and is influence by several factors. It has been reported that postoperative HCC recurrence is closely related to the biological tumor characteristics, including the number of tumors, tumor size, MVI, tumor capsule integrity, and degree of tumor cell differentiation (20, 21). The presence of cancer cell nests within the endothelial cell lining under a microscope was defined as MVI (22). Cancer cells may exist inside the hepatic or portal vein, thereby increasing the risk for intrahepatic and distant metastasis. Thus, MVI is an important cause of recurrence and poor prognosis in HCCs after surgical resection (23). Several studies have shown that MVI is an important indicator of early recurrence and survival rate after hepatectomy (10, 24, 25). Some scholars have suggested that, compared to Milan standards, MVI is a better predictor of recurrence (26). Studies have also demonstrated that tumor diameter is related to early postoperative recurrence in HCCs, the larger the tumor, the greater the risk of recurrence (18, 27, 28). Multiple tumors and tumors measuring > 5 cm are associated with a high risk of tumor recurrence and poor prognosis in postoperative HCC patients. This may be related to severe liver damage, high degree of sclerosis, and liver reserve function. Serum albumin level is an indicator of liver function. A recent study exploring the predictive value of serum albumin for recurrence after curative HCC resection found that a decrease in the preoperative serum albumin levels was significantly associated with a decrease in the overall survival and recurrence-free survival rate after radical HCC resection (29).

We constructed a prediction model based on the three independent risk factors for early recurrence of HBV-related HCCs after curative resection. The likelihood ratio test for the prediction model showed that it could predict early recurrence. To evaluate the quality of the prediction model, we evaluated the discrimination and calibration of the prediction model. Differentiation refers to the ability of a predictive model to identify early recurrence in HCC patients. Calibration is the consistency between the predicted and observed probabilities of early postoperative recurrence in HCC patients. We used calibration curves to examine the calibration ability of the prediction model. We collected the relevant case data for HBV-related HCC patients discharged from the hepatobiliary and pancreatic surgery departments of two hospitals during the same period after curative resection. These cases were used for external validation of the early recurrence prediction model by drawing ROCs and calibration curves. The results indicated that the prediction model had good differentiation and extrapolation.

This prediction model incorporates three of the most common clinical parameters, which makes it simple and easy to use. In addition, the predictive model is presented as a column chart, making it more concise and understandable. The model may be used by clinicians in their daily work to communicate more intuitively with patients in their daily work. In the current context of evidence-based and personalized medicine, for predicting high-risk populations for recurrence, necessary preventive measures can be taken (such as preventive TACE, antiangiogenesis therapy, PD-1/PD-L1, etc.) to delay HCC recurrence and improved the postoperative survival rate of these patients.

After HCC recurrence and metastasis, immunotherapy and antiangiogenesis therapy can complement each other and provide a comprehensive approach to managing the disease, especially in advanced stages or when the tumor is unresectable. Second-line treatments come into play when patients either do not respond to initial therapies or experience disease progression. There are many second-line targeted drugs, it is believe that how to choose suitable targeted drugs are a concern for readers, a meta-analysis suggest the use of regorafenib and cabozantinib as second-line treatments in HCC (30).

Limitations of the study: Our study was a retrospective cohort study, with retrospective data collection and potential biases. In the next step, we plan to conduct a multicenter prospective cohort study and explore the inclusion of more biomarkers or the combination of radiomics and pathomics to improve the predictive model, in order to further enhance its predictive ability. We are currently conducting relevant research work with data scientists from the Medical School of Taizhou University.

## Conclusions

The independent risk factors for early recurrence in HBV-related HCC patients after surgery were tumor diameter > 5 cm, albumin level < 35 g/L, and MVI. The prediction model based on the three clinical indicators could predict postoperative HCC recurrence with good discrimination, calibration and extrapolation.

## Data Availability

The original contributions presented in the study are included in the article/supplementary material. Further inquiries can be directed to the corresponding authors.
